# The Effects of In Situ Growth of SiC Nanowires on the Electromagnetic Wave Absorption Properties of SiC Porous Ceramics

**DOI:** 10.3390/ma18091910

**Published:** 2025-04-23

**Authors:** Jingxiong Liu, Genlian Li, Tianmiao Zhao, Zhiqiang Gong, Feng Li, Wen Xie, Songze Zhao, Shaohua Jiang

**Affiliations:** 1 Hunan Provincial Key Laboratory of Xiangnan Rare-Precious Metals Compounds and Applications, School of Chemistry and Environmental Science, Xiangnan University, Chenzhou 423000, China; lifeng214@xnu.edu.cn (F.L.); 1200004@hnust.edu.cn (W.X.); 2 Jiangsu Co-Innovation Center of Efficient Processing and Utilization of Forest Resources, International Innovation Center for Forest Chemicals and Materials, College of Materials Science and Engineering, Nanjing Forestry University, Nanjing 210037, China; 13938044332@163.com; 3 College of Liling Ceramic, Hunan University of Technology, Zhuzhou 412007, China; 15174470062@163.com (G.L.); 18574940920@163.com (Z.G.); virtue0489@163.com (S.Z.)

**Keywords:** SiC porous ceramics, SiCnws, electromagnetic wave absorption

## Abstract

In situ-grown SiC nanowires (SiCnws) on SiC porous material (SiCnws@SiC) were prepared using sol–gel and carbothermal reduction methods, which substantially improves the electromagnetic wave absorption property of composite material. The crystallinity and purity of SiCnws are the best when the sintering temperature is 1600 °C. When the ratio of the carbon source (C) to the silicon source (Si) is 1:1, SiCnws@SiC composite exhibits excellent electromagnetic wave absorption performance, the minimum reflection loss is −56.95 dB at a thickness of 2.30 mm, and the effective absorption bandwidth covers 1.85 GHz. The optimal effective absorption bandwidth is 4.01 GHz when the thickness is 2.59 mm. The enhancement of the electromagnetic wave absorption performance of SiCnws is mainly attributed to the increase in the heterogeneous interface and multiple reflection and scattering caused by the network structure, increasing dielectric loss and conduction loss. In addition, defects could occur during the growth of SiCnws, which could become the center of dipole polarization and increase the polarization loss of composite materials. Therefore, in situ growth of SiCnws on SiC porous ceramics is a promising method to improve electromagnetic wave absorption.

## 1. Introduction

With the technological progress and growing requirements of society, 5G and future communication technologies are developing rapidly, and the demand for the control and management of electromagnetic waves is becoming more and more urgent [1,2,3]. In this context, the importance of absorbing materials has become increasingly prominent because of their key roles in reducing interference, enhancing signal quality, and expanding coverage. In the military, absorbing materials are one of the key components of stealth technology. With the improvement of stealth performance, research on absorbing materials is also deepening. Therefore, research on high-performance absorbing materials to solve issues caused by electromagnetic waves has received massive attention [4,5,6].

Ferrite and magnetic metal are traditional absorbing materials, which have the advantages of high permeability, high saturation magnetization, low cost, simple preparation, etc. But their wide application is limited by problems such as a narrow effective wave-absorbing frequency band, high density, and poor stability [7]. In order to optimize the properties of absorbing materials, scientists have focused on novel materials, including carbon materials [8], transition metal sulfides materials [9], conducting polymer materials [10], metamaterials [11], etc. Among them, SiC is widely studied because of its high mechanical strength, good chemical stability, and strong oxidation resistance. However, the impedance matching between single crystal SiC and free space is difficult to balance, thus limiting its wave-absorption performance [12]. Electromagnetic properties are the response characteristics of materials under the action of external electromagnetic fields, mainly including dielectric characteristics, magnetic characteristics, and conductive characteristics. It has been demonstrated in research that different morphologies of SiC show magnificently different electromagnetic characteristics, like SiC particles [13], SiC foams [14], SiCnws [15], and SiC whiskers [16], each with its own electromagnetic characteristics. The electromagnetic properties of SiC particles are influenced by factors such as their size, shape, interparticle spacing, and contact state. Smaller particles exhibit a higher specific surface area and more pronounced surface effects, while larger particles and specific shapes primarily affect electromagnetic wave scattering. The spacing and contact state between particles govern electron hopping and conduction between them. By tuning particle size, morphology, concentration, and distribution, the electromagnetic properties of the material can be effectively tailored [13]. The high specific surface area of silicon carbide foam results in a significant proportion of surface atoms, giving rise to prominent surface states and interface effects. The low-density foam structure reduces its dielectric constant and improves impedance matching with air. The three-dimensional porous network structure enables multiple scattering and reflection of electromagnetic waves during propagation [14]. SiCnws exhibit significant electromagnetic anisotropy due to their high aspect ratio. At the nanoscale dimensions, quantum confinement effects emerge, which modify the electronic band structure and alter the density of states. These changes then influence the material’s dielectric properties and electromagnetic loss characteristics [15]. SiC fibers typically possess a continuous fibrous structure with well-ordered crystalline arrangement and minimal defects. This structural characteristic facilitates efficient electron transport along the fiber axis, leading to high electrical conductivity. Under electromagnetic wave irradiation, the fibers generate strong induced currents, which lead to significant electromagnetic energy dissipation through ohmic losses, thereby exhibiting excellent electromagnetic wave absorption and attenuation properties [16]. Among them, SiCnws have outstanding advantages in the field of electromagnetic wave absorption due to their excellent electromagnetic properties, high temperature resistance, and unique one-dimensional nanostructure [17,18]. Hu et al. [19] synthesized bamboo 3CSiC nanoparticles with high-density packing defects by the molten-salt-assisted calcination method. The Cole–Cole semicircle shows that SiCnws have multiple dielectric relaxation characteristics and can adjust the reflection loss (RL) value by controlling the defect density (when the RL value is lower than −10 dB, it indicates that 90% of the electromagnetic wave is absorbed). Experimental results show that at 13.52 GHz, when the thickness is 1.9 mm, the minimum reflection loss (RLmin) is −48.1 dB. Han et al. [20] proposed that SiCnw networks are conducive to extending the propagation distance of microwave and enhancing the resonance loss caused by alternating electromagnetic field. This phenomenon can be attributed to the three-dimensional net structure formed by high-specific-surface-area SiCnws, which improves the interface characteristics, like dipolarization, multiple reflection, electromagnetic scattering, etc., and significantly enhances the absorption ability of electromagnetic waves [21,22,23]. The traditional single material has the problems of impedance mismatch, low conductivity, and a single loss mechanism in electromagnetic wave absorption performance [24,25]. To solve these problems, Wei et al. [26] prepared Re-SiC ceramics (Re: Sc, Y, Ce) through the PDCs method, whose data shows that the effective absorption bandwidth (EAB) and the thickness of Sc-SiC ceramics are, respectively, 3.2 GHz and 2.9 mm, and that the RLmin of the X-band is −10.8 dB. The results show that a suitable three-dimensional network structure can significantly improve the impedance matching characteristics and dielectric loss of Sc-SiC ceramics and thus obtain excellent microwave absorption performance. Su et al. [27] grew SiCnws on the surface of carbon fibers using polymer infiltration and pyrolysis methods. Also, they prepared a SiC matrix through the chemical vapor infiltration method. In this experiment, after three times of filtration, the bending strength of the composite material reaches 107.35 ± 10 MPa. When the material’s thickness is at 1.86 mm, the RLmin is −41.08 dB and the EAB (RL ≤ −10 dB) reaches 3.86 GHz. Therefore, the introduction of SiCnws into a porous ceramic matrix is a feasible method to improve the electromagnetic wave absorption efficiency of composite materials.

The sol–gel and carbothermal reduction method is a method using silicon dioxide gel containing carbon sources to prepare SiCnws. This method is simple and easy to use, has low process difficulty, and is suitable for the preparation of materials in large quantities. The sol–gel process can control the molar mass ratio of carbon to silicon in the reaction system and ensure the uniform mixing of the carbon source and the silicon source [28,29]. Meng et al. [30] prepared sucrose-containing silica sol by the sol–gel method. They used ethyl orthosilicate, anhydrous ethanol, sucrose, and deionized water as raw materials and HNO_3_ as a catalyst. SiC nanorods with a diameter of 15–30 nm and length of 20 μm were prepared by carbothermal reduction of SiO_2_ dry gel. Zhang et al. [31] synthesized SiOC xerogel using silica sol and sucrose as raw materials and obtained β-SiC nanowires at 1500 °C, in which the yield of SiC nanowires was 59%. The formation of crystal nuclei during the preparation of SiCnws by the sol–gel and carbothermal reduction method is related to gas-phase supersaturation: at lower temperatures, supersaturation is too small to form stable crystal nuclei, and the conversion rate of SiO_2_ and yield of SiCnws are low. When the temperature is too high, supersaturation is too large, conducive to the formation of SiC particles but not to that of SiCnws [32]. The sintering temperature has a significant effect on the growth of SiCnws. Subsequently, the growth situation of SiCnws at different temperatures will be discussed.

The growth mechanism of SiCnws includes a gas–liquid–solid mechanism (VLS) and gas–solid mechanism (VS). The main difference between the two is that the VLS mechanism introduces a catalyst. The introduction of a catalyst is beneficial to reduce the synthesis temperature of nanowires and accelerate the growth rate. In the growth process of SiCnws, the interface between catalyst droplets and solid materials follows the principle of minimum energy, promotes the growth of anisotropic crystals, and preferentially grows under the catalysis of catalyst droplets to obtain nanowires [27]. By controlling the size and shape of the catalyst particles, the cross-section of the nanowires can be adjusted, thus controlling the microstructure of the nanowires. Duan et al. [33] synthesized SiCnw-containing SiOC ceramics [34]. They pyrolyzed polysiloxane catalyzed by ferrocene particles at an annealing temperature of 1350–1450 °C. With the increase in annealing temperature and ferrocene content, the content of SiCnws increases gradually, the real dielectric constant and imaginary dielectric constant of the ceramics increase from 3.63 and 0.14 to 10.72 and 12.17, respectively, and the minimum reflection coefficient decreases from −1.22 dB to −20.01 dB. Wei et al. [35] synthesized Carbon@SiC(SiCnws)-Sc_2_Si_2_O_7_ ceramics by H_2_ reduction and CVI technology, and they used nickel particles as metal catalysts to regulate the growth of SiCnws. In the X-band, the total shielding effectiveness of the composite ceramic with a thickness of 2 mm is −31.8 dB, indicating 99.92% electromagnetic wave attenuation. Therefore, not only the addition of the catalyst contributes to the growth of nanowires, but doping transition metal particles also helps to improve the dielectric properties of the composite materials.

Compared with other electromagnetic wave-absorbing materials, SiC-based electromagnetic wave-absorbing materials have unique advantages in all aspects. Compared with metal-based electromagnetic wave-absorbing materials, SiC-based materials have good chemical stability; compared with polymer-based materials, SiC-based materials have excellent high-temperature stability; in addition, SiC-based electromagnetic wave-absorbing materials also have excellent thermal shock resistance, which can not only be applied in high-temperature environments but also in harsh and harsh environments [36]. Combined with previous relevant research, it was found that the dielectric loss capacity in a material depends mainly on the relative complex dielectric constant of the material reference. There are many factors that affect the relative complex dielectric constant, the most important of which are the morphology and microstructure of the material itself [37]. It was found that different morphologies of SiC can be applied in electromagnetic wave absorption fields, such as SiC ceramics, SiC powders, SiC fibers, and SiCnws. Currently, these materials usually absorb electromagnetic waves as coatings or dense structures, but this can relatively increase the density of the material [23]. At the same time, single-phase SiC has one disadvantage as an electromagnetic wave-absorbing material: the effective absorption bandwidth is narrow [38]. To address these limitations, one effective strategy involves fabricating porous SiC structures and incorporating secondary phases (e.g., SiC nanowires) through loading or doping. This approach enhances interface polarization loss, thereby improving the electromagnetic wave absorption capabilities of SiC-based materials [39]. However, current studies on electromagnetic wave absorption materials of SiC porous ceramics are relatively scarce. Therefore, further in-depth exploration of the impact of SiC porous ceramics on the properties of electromagnetic wave absorption materials is still a key topic that needs to be continuously studied in this field [40].

In this paper, SiCnws were grown in situ on SiC porous ceramic materials by the sol–gel and carbothermal reduction method. The internal relationship between the sintering temperature and carbon–silicon ratio and the structure of SiCnws was studied in detail, and the influence of the microstructure on the electromagnetic wave absorption properties of composite ceramic materials was investigated.

## 2. Materials and Methods

### 2.1. Materials

Glucose (analytically pure, Sinopharm Chemical Reagent Co., Ltd., Shanghai, China), citric acid (analytically pure, Sinopharm Chemical Reagent Co., Ltd., Shanghai, China), ethyl orthosilicate (analytically pure, Tianjin Kemiou Chemical Reagent Co., Ltd., Tianjin, China), nickel sulfamate tetrahydrate (analytically pure, Shanghai McLean Biochemical Technology Co., Ltd., Shanghai, China), and SiC powder (coarse powder: 150 μm, fine powder: 200 nm, purity 99%, Changle Hongxin Grinding Material Co., Ltd., Weifang, China) were obtained.

### 2.2. Preparation Process

#### 2.2.1. Preparation of Matrix

First, a SiC mixture with a 3:1 mass ratio of coarse powder to fine powder was added together with an appropriate amount of 10 wt% PVA in a mortar. After mixing uniformly by stirring for 1 h, the obtained mixture was transferred into a mold. A pressure of 10 MPa was applied and maintained for 30 s to press the powder into a test-block with dimensions of 22.86 mm × 10.16 mm × 3 mm. Then, the test-block was dried at 100 °C for 24 h. Finally, the obtained dried sample was placed in a tube furnace filled with an argon atmosphere. The sample was sintered at 2400 °C for 2 h to finally obtain the porous SiC matrix.

#### 2.2.2. Preparation of SiCnws

Firstly, the SiC precursor was prepared by the sol–gel method. Glucose was used as the carbon source, ethyl orthosilicate as the silicon source, and nickel sulfamate tetrahydrate as the catalyst. Aqueous glucose as the carbon source and ethanol-dissolved tetraethyl orthosilicate as the silicon source were mixed at a 1:1 mass ratio. After mixing, nickel aminosufonate (accounting for 10 wt% of the total mass of the carbon source and silicon source) and citric acid (to adjust the pH to 4–5) were added to form a homogeneous sol.

Secondly, the prepared matrix was immersed in the sol for 30 min in a vacuum state and dried at 90 °C for 12 h. This process was repeated 3 times.

Finally, the dried samples were sintered in an argon atmosphere in a tube furnace. The heating rate was 5 °C/min; the holding time was 4.5 h; and the sintering temperatures were 1400 °C, 1450 °C, 1500 °C, 1550 °C, 1600 °C, and 1650 °C, respectively.

**Figure 1:**
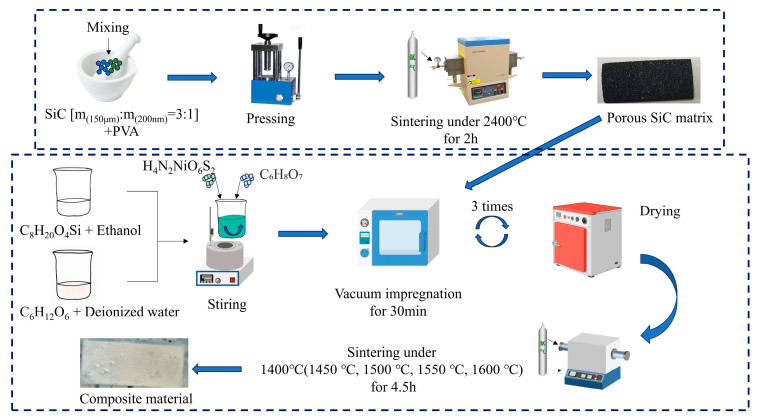
Preparation process of SiCnws@SiC.

The SiC precursor with C-to-Si ratios of 1:2 and 2:1 were, respectively, configured using the same steps above, and then the SiC porous matrix was impregnated and sintered at 1600 °C to prepare SiCnws@SiC composite material. The preparation process of SiCnws@SiC composite material is shown in Figure 1.

### 2.3. Characterization

The phase composition of the composites was analyzed by X-ray powder diffraction (XRD, Rigaku D/max 2550, Cu Kα radiation, Osaka, Japan). The working parameters were as follows: tube voltage, 40 kV; tube current, 30 mA; scanning angle range, 5°~80°; sampling interval, 0.02°; scanning speed, 5°/min. The morphology and microstructure were analyzed by field emission scanning electron microscopy (SEM, Tescan Mira4, Shanghai, China). Before measurements, the samples were sprayed with gold to enhance conductivity and SEM image quality. The sample elements were scanned using an X-ray dispersive spectrometer (EDS) equipped with an SEM, and qualitative and semi-quantitative analysis was performed on the sample micro-region elements. The variable temperature electromagnetic parameters in the range of 8.2–12.4 GHz were measured by the Agilent N5230A (Shenzhen, China) vector network analyzer.

## 3. Results and Discussion

### 3.1. The Effect of Temperature on the Morphology of SiCnws

**Figure 2:**
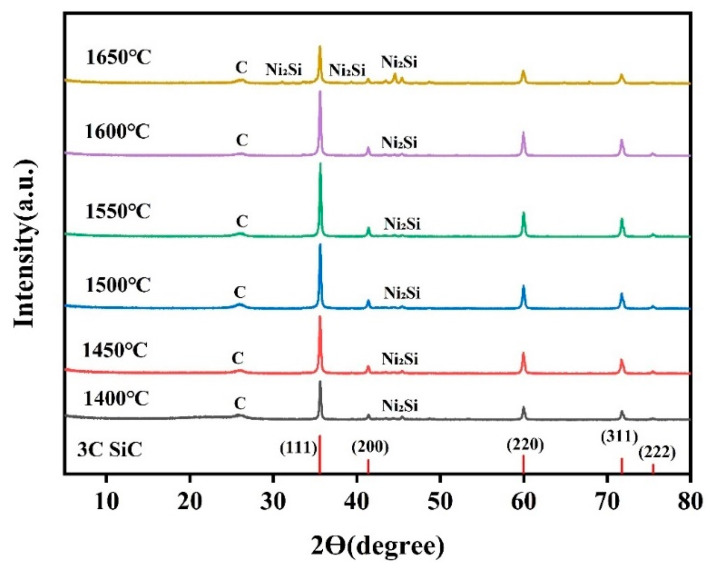
XRD patterns of precursor powders at different sintering temperatures.

In order to explore the optimal growth temperature of SiCnws, the prepared SiC precursor powders were sintered at 1400 °C, 1450 °C, 1500 °C, 1550 °C, 1600 °C, and 1650 °C, respectively, and their phase composition was analyzed. The phase composition and crystal structure materials can be characterized by XRD analysis [41,42,43]. Figure 2 is the XRD pattern of the precursor powder at different temperatures. The XRD pattern shows that the sample mainly contains SiC. With the increase in sintering temperature, the characteristic peak of SiC increases first and then decreases, and the characteristic peak of carbon gradually decreases. The characteristic peaks of SiC appear at 35.73°, 41.49°, 60.13°, 71.95°, and 75.69°, corresponding to the (111), (200), (220), (311), and (222) crystal plane of SiC (NO. 73-1665). According to PDF card NO. 73-1665, the type of SiCnws is 3C-SiC, which belongs to the cubic crystal system. Cubic SiCnws have better microwave absorption performance [27]. At 1600 °C, the characteristic peaks of carbon almost disappear, showing the excellent crystallinity and purity of silicon carbide. There is also a Ni_2_Si peak in the XRD pattern. As nickel atoms reduce the reaction activation energy to promote the reaction of carbon source and silicon source, nickel atoms can also interact with silicon atoms to form Ni_2_Si, which is due to a certain chemical affinity between nickel and silicon atoms [44].

**Figure 3:**
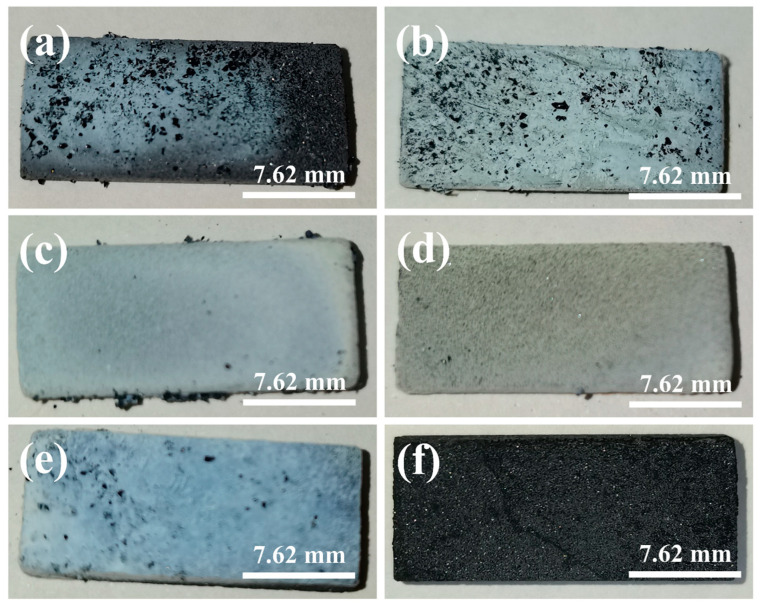
Photoes of SiCnws@SiC composites at different sintering temperatures: (**a**) 1400 °C, (**b**) 1450 °C, (**c**) 1500 °C, (**d**) 1550 °C, (**e**) 1600 °C, and (**f**) 1650 °C.

**Figure 4:**
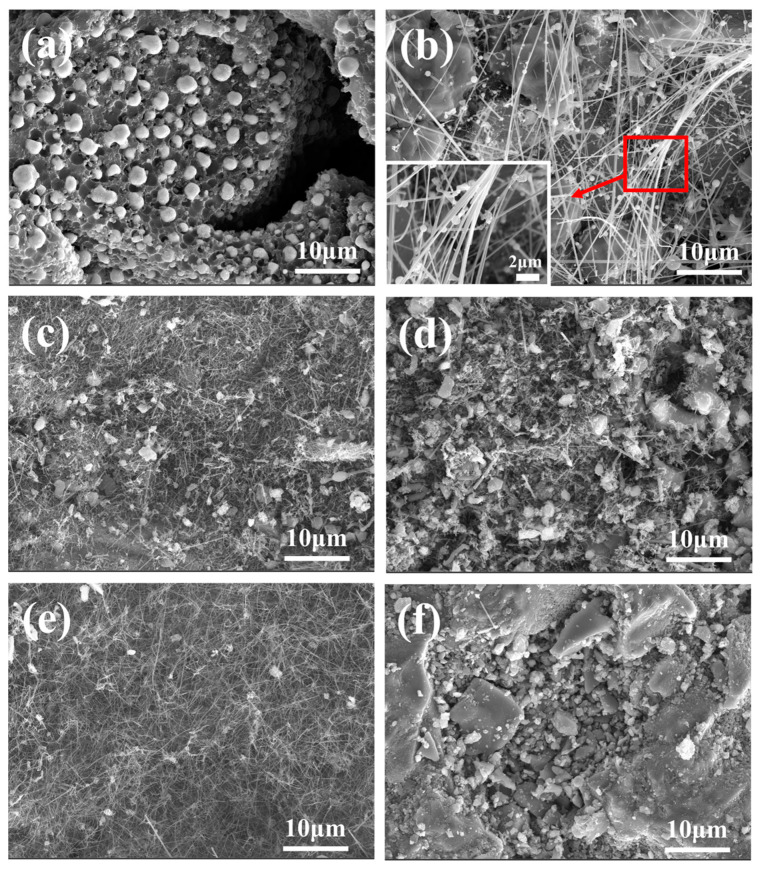
Micromorphology of the outer surface of SiCnws@SiC composites at different temperatures: (**a**) 1400 °C, (**b**) 1450 °C, (**c**) 1500 °C, (**d**) 1550 °C, (**e**) 1600 °C, and (**f**) 1650 °C.

**Figure 5:**
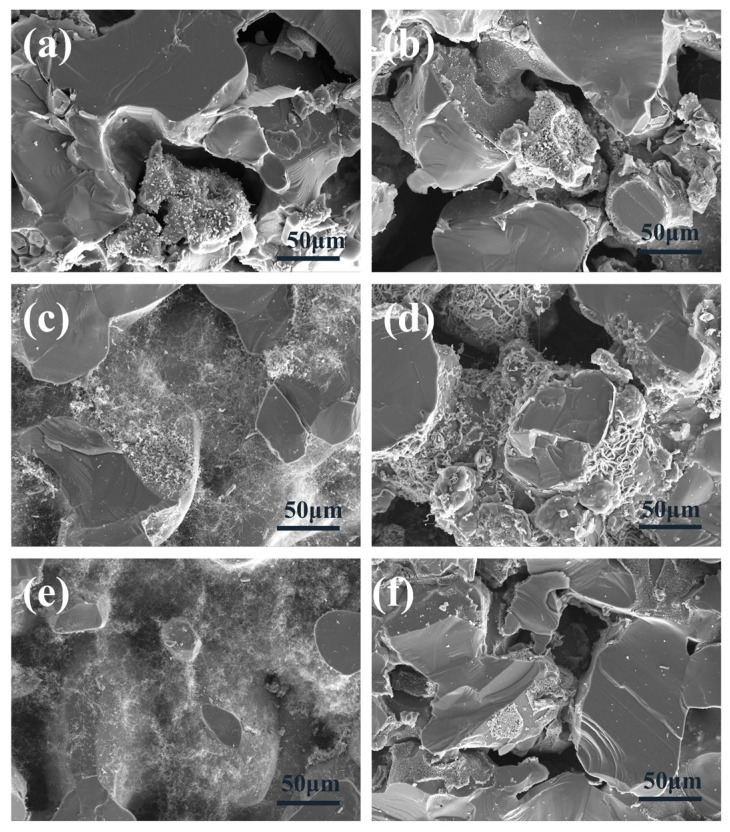
Micromorphology of the inner surface of SiCnws@SiC composites at different temperatures: (**a**) 1400 °C, (**b**) 1450 °C, (**c**) 1500 °C, (**d**) 1550 °C, (**e**) 1600 °C, and (**f**) 1650 °C.

**Figure 6:**
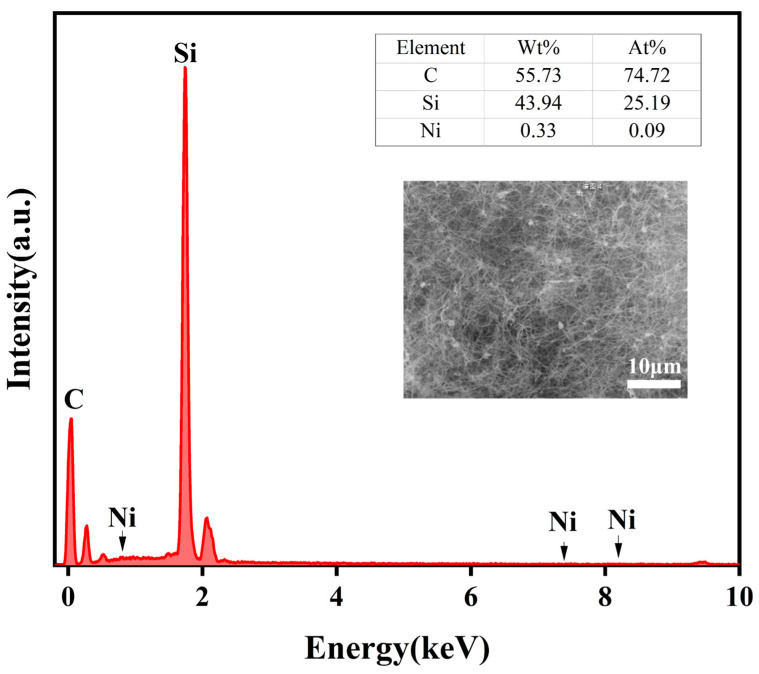
X-ray energy spectrum analysis of nanowires.

In order to further determine the sintering temperature, the morphology and microstructure of the samples were characterized by SEM [45,46]. Figure 3 shows the morphology of the sample at different sintering temperatures. According to Figure 3a, at 1400 °C, white cotton-like substances are formed on the surface of the substrate, but only in some areas, and the distribution is not uniform. As shown in Figure 3b–e, the whole matrix is covered by white cotton-like substances at other temperatures. From Figure 3f, the surface of the sample was observed to have no cotton-like substances at a temperature of 1650 °C. The micromorphologies of materials can be observed by SEM images [47,48]. Figure 4a–f is the micromorphology of the outer surface of the sample at 1400 °C, 1450 °C, 1500 °C, 1550 °C, 1600 °C, and 1650 °C, respectively. Figure 5a–f is the micromorphology of the inner surface of the sample at the different temperatures above, respectively. As shown in Figure 4a and Figure 5a, nanoparticles appeared on the inner and outer surfaces of the sample at 1400 °C, but no nanowires were observed; the temperature increased by 50 °C, and nanowires were observed on the outer surface of the sample (Figure 4b), but only a very few nanowires were observed on the inner surface of the sample (Figure 5b). At 1500 °C, 1550 °C, and 1600 °C, a large number of nanowires were observed on the outer surface and inner surface of the sample, and the growth state of the nanowires was relatively uniform at 1600 °C. At 1650 °C, nanowires were observed on the inner and outer surfaces of the sample, and the grains grew transversely due to the increase in temperature. X-ray energy spectrum analysis (EDS) can be effectively applied to measure the elemental composition of materials [49,50,51]. As shown in Figure 6, it shows that the element composition of nanowires is carbon, silicon, and nickel. Combined with XRD pattern analysis, it can be seen that the phase of the nanowire is SiC. Figure 4b shows that there are spherical particles at the end of the nanowires. According to the X-ray energy spectrum, the nanowires are SiCnws, the spherical particles are nickel droplets, and the nickel droplets are used as the growth point of the nucleation of SiCnws, indicating that the growth of SiCnws conforms to the gas–liquid–solid growth mechanism. The interface between the catalyst alloy droplets and the solid material follows the minimum energy principle, promotes the growth of anisotropic crystals, and preferentially grows under the catalysis of the catalyst droplets to obtain nanowires [12]. Comparing the microtopography at different temperatures, the growth of SiCnws on the inner and outer surfaces of the matrix is more uniform at 1600 °C. Therefore, the synthesis temperature of SiCnws is 1600 °C in the subsequent preparation of composite materials.

### 3.2. Morphology and Micromorphology of Composites

The porosity (P) of SiC porous ceramics was measured by the Archimedes drainage method. First, the SiC porous ceramics were placed in boiling water for one hour to measure the wet weight (*m*_3_) and suspended weight (*m*_2_); secondly, the sample was placed in an oven, dried of all moisture, and measured for its dry weight (*m*_1_); finally, the porosity of the sample was calculated by Formula (1). The pore size of the sample was tested using a microfiltration membrane pore size analyzer (Shanghai Lichenbangxi Instrument Technology Co., Ltd., Shanghai, China), and the results are shown in Table 1. From the table, it can be seen that the porosity of silicon carbide porous ceramics is 30% on average.

(1) $$P=\frac{m_{3}-m_{1}}{m_{3}-m_{2}}\times 100\%$$

**Figure 7:**
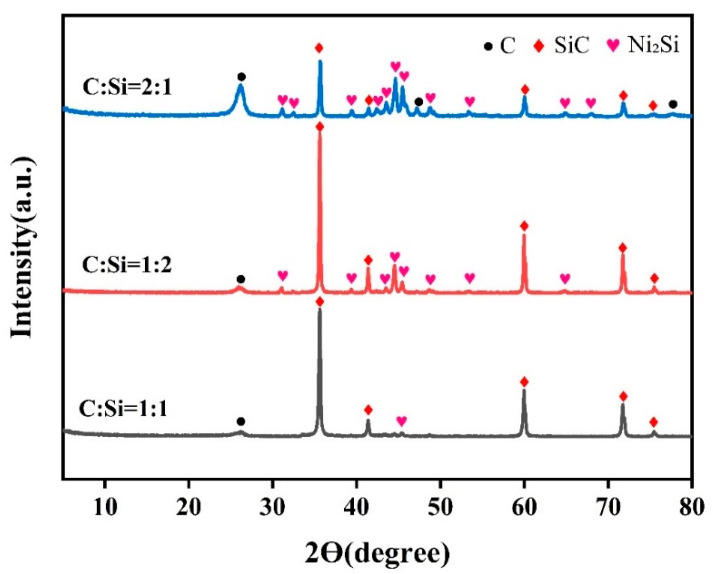
XRD patterns of precursor powders with different C to Si ratio at 1600 °C.

After determining the sintering temperature, the optimal carbon and silicon source ratio for the growth of SiCnws was explored. Figure 7 shows the XRD pattern of the precursor powders with different carbon-to-silicon ratios sintered at 1600 °C. It can be seen from the figure that when the C:Si ratio is 1:1, the sample has fewer impurity peaks, mainly SiC peaks. Compared with the case when the C:Si ratio is 1:1, the SiC peak in the sample is stronger when the C:Si ratio is 1:2, but with the increase in the silicon source, more Ni_2_Si peaks appear in the sample, and the purity of the sample decreases. When the C:Si ratio is 2:1, the SiC peak in the sample is significantly lower than the other two ratios. As the carbon source increases, the C peak in the sample increases. Therefore, excellent SiC crystallinity and purity are shown when the C:Si ratio is 1:1.

**Figure 8:**
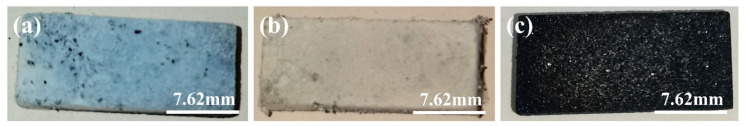
Photographs of SiCnws@SiC composites with different C to Si ratios at 1600 °C: (**a**) C:Si = 1:1, (**b**) C:Si = 1:2, and (**c**) C:Si = 2:1.

**Figure 9:**
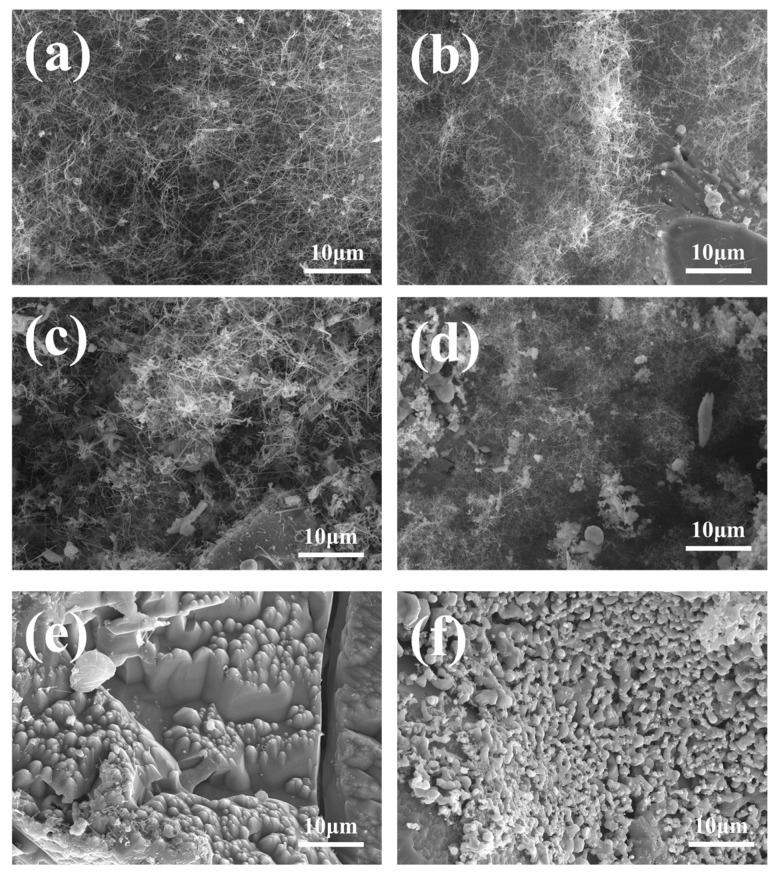
Micromorphology of SiCnws@SiC composites with different C to Si ratios at 1600 °C: (**a**,**b**) C:Si = 1:1, (**c**,**d**) C:Si = 1:2, and (**e**,**f**) C:Si = 2:1; (**a**,**c**,**e**) outer surface; (**b**,**d**,**f**) inner surface.

Figure 8 shows the samples sintered at different C-to-Si ratios at 1600 °C. When the C:Si ratios are 1:1 and 1:2, the surface of the sample is covered with white cotton-like substances, and no white cotton-like substances are observed in the sample with a C:Si ratio of 2:1. In order to further confirm the C-to-Si ratio, the micromorphology of the sample was characterized. Figure 9 is the micromorphology of SiCnws@SiC composites with different C: Si ratios at 1600 °C. Figure 9a,c, and e are the micromorphology of the outer surface of samples with a C:Si ratio of 1:1, C:Si ratio of 1:2, and C:Si ratio of 2:1, respectively. Figure 9b,d,f are the micromorphology of the inner surface of the above proportions, respectively. When the C:Si ratio is 1:1, a large number of slender SiCnws are observed on the inner and outer surfaces of the sample; large-diameter SiCnws are observed in samples with a C:Si ratio of 1:2. When the C:Si ratio is 2:1, only a small number of SiCnws are observed on the surface of the sample, and the surface pores are blocked, while large-diameter SiCnws appear on the inner surface. Generally, SiCnws with smaller diameters are conducive to improving electromagnetic wave absorption performance. This is because a smaller diameter can increase the specific surface area of the nanowire, thereby enhancing the scattering and absorption of electromagnetic waves; a smaller diameter can increase the imaginary part of the dielectric constant, which means that the material’s polarization loss ability to electromagnetic waves is enhanced, thereby improving the wave absorption performance. Combined with XRD pattern analysis, the growth of SiCnws in the sample with a C:Si ratio of 1:1 meets the requirements.

### 3.3. Dielectric Properties

SiC ceramic material is non-magnetic ($\mu^{\prime }=1$, $\mu^{\prime \prime }=0$), so its electromagnetic wave absorption performance mainly depends on the complex dielectric constant [23]. The complex dielectric constant ($\varepsilon_{r}=\varepsilon^{\prime }-j\varepsilon^{\prime \prime }$) and loss tangent ($tan\;\delta =\varepsilon^{\prime \prime }/\varepsilon^{\prime }$) are two key factors that determine the electromagnetic wave absorption properties of materials. The real part of the complex dielectric ($\varepsilon^{\prime }$) represents the storage capacity of the material for electromagnetic wave energy, and the imaginary part of the complex dielectric ($\varepsilon^{\prime \prime }$) represents the loss capacity of the material for electromagnetic wave energy. The tan δ is one of the key parameters to evaluate the absorption and attenuation characteristics of electromagnetic waves. The larger the imaginary part of the complex dielectric, the higher the loss tangent value, the larger the dielectric loss, and the stronger the electromagnetic wave loss ability of the material [52,53].

**Figure 10:**
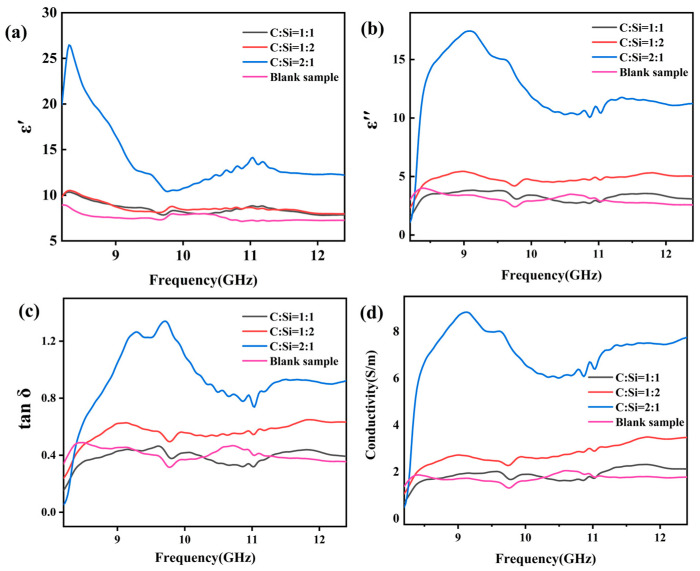
Complex permittivity of SiCnws@SiC composites: (**a**) real permittivity, (**b**) imaginary permittivity, (**c**) loss angle, and (**d**) conductivity.

Figure 10 shows the complex permittivity and conductivity of SiCnws@SiC composites in the frequency range of 8.2–12.4 GHz. With the increase in frequency, the $\varepsilon^{\prime }$ value shows a downward trend and is accompanied by a certain fluctuation, which is due to the frequency scattering effect as well as the formation of interfaces and dipoles induced by nanoparticles, resulting in a lag in the frequency-scattering effect under alternating electromagnetic fields. The average $\varepsilon^{\prime }$ values of the three samples are 8.5, 8.6, and 14.1, respectively, indicating that the sample with a C:Si ratio of 2:1 has a strong microwave storage capacity compared with other samples. The average $\varepsilon^{\prime }$ values of the three samples are 3.3, 4.8, and 12.4, respectively. The $\varepsilon^{\prime \prime }$ curves of the samples have resonance peaks near 9, 9.5, and 11 GHz, which are caused by dipole rotation-induced dipole polarization and interface polarization caused by a heterogeneous interface [37]. The average tan δ values are 0.39 (C:Si ratio of 1:1), 0.56 (C:Si ratio of 1:2), and 0.93 (C:Si ratio of 2:1), indicating that the sample with a C:Si ratio of 2:1 has high dielectric loss performance. According to Debye theory, $\varepsilon^{\prime }$ and $\varepsilon^{\prime \prime }$ can be expressed by specific expressions, as follows in (2) and (3) [23,54,55].

(2) $$\;\varepsilon^{\prime }=\varepsilon_{\infty }+\left(\varepsilon_{s}-\varepsilon_{\infty }\right)\;/\;\left(1+{\left(\omega \tau \right)}^{2}\right)$$

(3) $$\varepsilon^{\prime \prime }=\left(\varepsilon_{s}-\varepsilon_{\infty }\right)\;\omega \tau \;/\;\left(1+{\left(\omega \tau \right)}^{2}\right)+\sigma \;/\;\omega \varepsilon_{0}$$

Among these $\varepsilon_{\infty }$ is the optical frequency dielectric constant; $\varepsilon_{s}$ is the static dielectric constant, ω is the angular frequency; and τ, σ and $\varepsilon_{0}$ are the polarization relaxation time, conductivity, and free space dielectric constant, respectively.

According to Equations (2) and (3), the value of the real part of the complex permittivity is mainly related to the polarization phenomenon, and the imaginary part of the complex permittivity is positively correlated with the conduction loss [56]. Compared with the unsintered sample, the complex dielectric constant and loss tangent have a certain degree of increase. The addition of SiCnws increases the heterogeneous interface, and the interfacial polarization between the interface and the interface increases greatly, resulting in an increase in the complex dielectric real part of the composite. Figure 10d shows the electrical conductivity of SiCnws@SiC composites. SiC is a wide-band gap semiconductor, and one-dimensional SiCnws have unique electrical properties. Therefore, the addition of SiCnws can affect the conductivity of the composites. The residual C phase of the grown SiCnws can also increase the conductivity of the composites. The conductivity of the composite material increases, and the imaginary part of the complex dielectric also increases accordingly. Moreover, the network structure formed by the SiCnws increases the transmission channel of the carrier and further improves the complex dielectric imaginary part of the composite material.

The conductivity in the material can affect the conduction loss, which also has a certain effect on the dielectric loss. The greater the conductivity of the material, the greater the conduction loss [57]. The conductivity of the sample with a C:Si ratio of 2:1 is the largest, indicating that the conductivity loss of the sample is better, but the high conductivity can lead to the impedance mismatch of the material [35].

There are multiple resonance peaks in the ε″ curve, indicating that there are multiple polarization relaxation processes. The Cole–Cole semicircle can be used to analyze the polarization relaxation process of the composite material. According to Equations (2) and (3), the Cole–Cole equation can be derived as follows in (4) [58].

(4) $${\left(\varepsilon^{\prime }-\frac{\varepsilon_{S}+\varepsilon_{\infty }}{2}\right)}^{2}+{\left(\varepsilon^{\prime \prime }\right)}^{2}={\left(\frac{\varepsilon_{S}-\varepsilon_{\infty }}{2}\right)}^{2}$$

**Figure 11:**
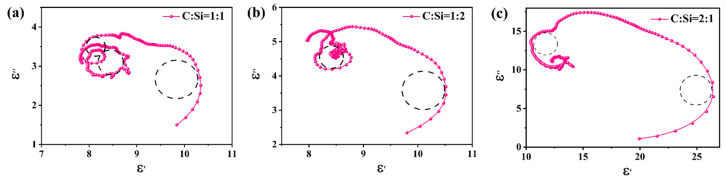
Cole–Cole curve of SiCnws@SiC composites: (**a**) C:Si = 1:1, (**b**) C:Si = 1:2, and (**c**) C:Si = 2:1.

When the relationship between $\varepsilon^{\prime }$ and $\varepsilon^{\prime \prime }$ can be described as a semicircle, it can be considered a Cole–Cole semicircle. Each Cole–Cole semicircle can represent a relaxation mechanism. The Cole–Cole diagram of the composite material is shown in Figure 11. Three semicircles can be observed in the sample with a C:Si ratio of 1:1, representing three relaxation mechanisms, and two semicircles in the samples with a C:Si ratio of 1:2 and C:Si ratio of 2:1. Compared with other samples, the sample with a C:Si ratio of 1:1 showed more semicircles, indicating that the polarization relaxation process is enhanced. When SiCnws are added, a large number of defects and nanoheterogeneous interfaces can be formed. Defects can act as the center of dipole polarization, and nanoheterogeneous interfaces have interfacial polarization [12], such as SiCnws/SiC nanoheterogeneous interfaces. The 3D network structure formed by SiCnws and composites can produce cross-polarization, which also further contributes to the Debye relaxation process [26].

### 3.4. Electromagnetic Wave Absorption Properties

Impedance matching and the attenuation constant are two important parameters in the design of electromagnetic wave-absorbing materials. High impedance matching can minimize the reflection of electromagnetic waves on the surface of the material, so that as many electromagnetic waves as possible enter the material. In general, when the impedance matching of the ideal electromagnetic wave-absorbing material is close to 1, the surface reflection is less when the electromagnetic wave enters the material, and the impedance matching can be calculated according to Equation (5). The high attenuation constant indicates that the material can effectively convert the electromagnetic wave energy into heat energy or other forms of energy, thereby reducing the propagation depth of the electromagnetic wave inside the material. The attenuation constant can be calculated from Equation (6) [53,56].

(5) $$Z=\left|Z_{in}/Z_{0}\right|=\sqrt{\frac{\mu_{r}}{\varepsilon_{r}}}\tanh\left[j\frac{2\pi fd}{c}\sqrt{\mu_{r}\varepsilon_{r}}\right]$$

(6) $$\alpha =\frac{\sqrt{2}\pi f}{c}\sqrt{(\mu^{\prime \prime }\varepsilon^{\prime \prime }-\mu^{\prime }\varepsilon^{\prime })+\sqrt{{\left(\mu^{\prime \prime }\varepsilon^{\prime \prime }-\mu^{\prime }\varepsilon^{\prime }\right)}^{2}+{\left(\mu^{\prime }\varepsilon^{\prime \prime }-\mu^{\prime \prime }\varepsilon^{\prime }\right)}^{2}}}$$

**Figure 12:**
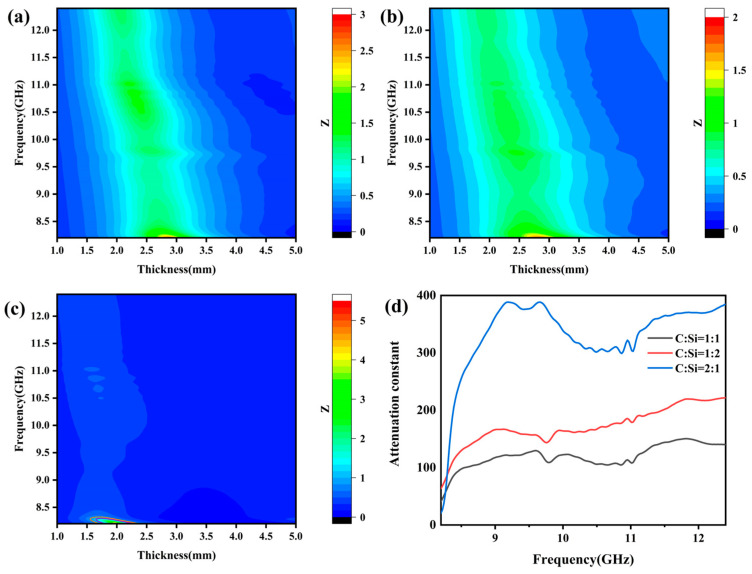
Two-dimensional figure of Z value of SiCnws@SiC composites with different thickness changing with frequency: (**a**) C: Si = 1:1, (**b**) C: Si = 1:2, (**c**) C: Si = 2:1, and (**d**) attenuation constant.

Figure 12 shows the impedance matching and attenuation constant of SiCnws@SiC composites. The sample with a C:Si ratio of 2:1 has the highest attenuation constant, and the other two samples have little difference in average attenuation constant. From the two-dimensional impedance matching diagram of the sample, it can be clearly observed that the frequency range of the Z value of the sample with a C:Si ratio of 1:1 near 1 is larger than those of the samples with C:Si ratios of 1:2 and 2:1, and the sample with a C:Si ratio of 2:1 only has impedance matching in a very small part of the region. The attenuation constant of the sample with a C:Si ratio of 2:1 is the largest among the three samples, but its impedance mismatch causes the electromagnetic wave to fail to enter the material and be attenuated. Only when the two conditions are met at the same time can the material show good electromagnetic wave absorption performance.

Reflection loss (RL) is an important parameter to characterize the performance of absorbing materials. When the reflection loss value is lower than −10 dB, it means that 90% of the electromagnetic wave is absorbed, and the frequency band in this range is the effective absorption bandwidth (EAB). According to the transmission line theory, RL can be calculated from the measured values of complex permittivity and permeability. The equation is as follows (7)–(9) [52,59]:

(7) $$RL(dB)=20\mathrm{lg}\left|\frac{Z_{in}-Z_{0}}{Z_{in}+Z_{0}}\right|$$

(8) $$Z_{in}=Z_{0}\sqrt{\frac{\mu_{r}}{\varepsilon_{r}}}\tanh\left[j\frac{2\pi \;fd}{c}\sqrt{\mu_{r}\varepsilon_{r}}\right]$$

(9) $$Z_{0}=\sqrt{\frac{\mu_{0}}{\varepsilon_{0}}}$$

In the equation, $Z_{in}$ and $Z_{0}$ represent the input impedance and free space impedance, respectively; f, d, and c are the average frequency of light, the thickness of the sample, and the vacuum velocity of light, respectively. $\varepsilon_{r}=\varepsilon^{\prime }-j\varepsilon^{\prime \prime }$ and $\mu_{r}=\mu^{\prime }-j\mu^{\prime \prime }$ represent the complex permittivity and complex permeability of the material.

**Figure 13:**
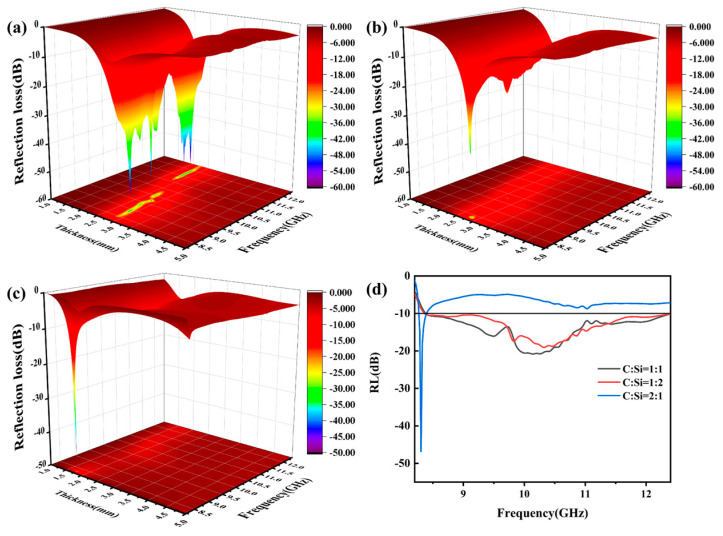
Two-dimensional figure of RL value of SiCnws@SiC composites with different thickness changing with frequency: (**a**) C:Si = 1:1, (**b**) C:Si = 1:2, (**c**) C:Si = 2:1, and (**d**) EAB.

Figure 13 shows the change in the reflection loss of SiCnws@SiC composites with frequency under different thicknesses. The minimum RL (RLmin) of the sample with a C:Si ratio of 1:1 is −56.95 dB when the thickness is 2.30 mm, and the EAB covers 1.85 GHz. The RLmin of the sample with a C:Si ratio of 1:2 is −38.61 dB when the thickness is 2.83 mm, and the EAB covers 2.52 GHz. The RLmin of the sample with a C:Si ratio of 2:1 is −46.84 dB when the thickness is 1.77 mm, and the EAB is only 0.11 GHz. The optimal EAB of SiCnws@SiC composites in the test frequency band is as follows: The optimal EAB of the sample with a C:Si ratio of 1:1 is 4.01 GHz when the thickness is 2.59 mm. The optimal EAB of the sample with a C:Si ratio of 1:2 when the thickness is 2.50 mm is 4.03 GHz. The optimal EAB of the sample with a C:Si ratio of 2:1 when the thickness is 1.77 mm is 0.11 GHz. In summary, compared with the other two samples, the sample with a C:Si ratio of 1:1 has better electromagnetic wave absorption performance. The sample with a C:Si ratio of 1:1 not only has the maximum RLmin value but also has a wider EAB when the sample’s thickness has no big difference.

### 3.5. Electromagnetic Wave-Absorbing Mechanism

**Figure 14:**
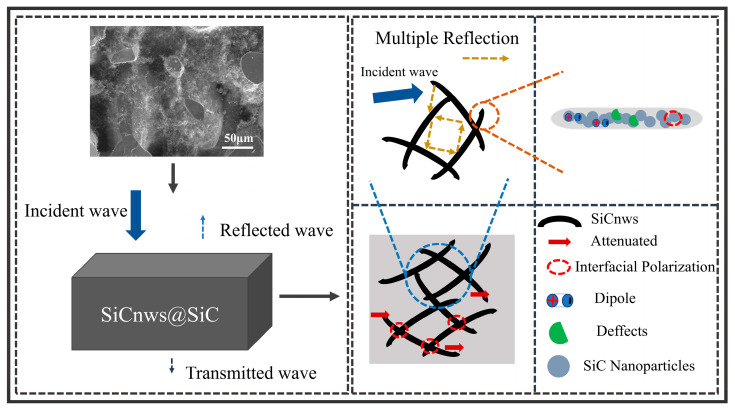
Electromagnetic wave absorption mechanism of SiCnws@SiC composite ceramics.

Figure 14 shows the electromagnetic wave absorption mechanism of SiCnws@SiC composites. When the electromagnetic wave is incident on the composite, part of it is reflected and transmitted, and most of it is absorbed into the material. First, the addition of SiCnws improves the impedance of the composite material, forming a structure with gradual impedance matching characteristics, so that electromagnetic waves can effectively enter the composite material, thereby increasing the contact area between electromagnetic waves and SiCnws [60]. The network structure formed by SiCnws and ceramic composite materials will cause multiple reflections and scattering of incident electromagnetic waves, so that the incident wave is attenuated [60,61]. Secondly, defects will be generated during the growth of SiCnws, and the defects will become the center of dipole polarization, thereby improving the polarization loss [26]. The network structure will also increase the transmission channel of carriers. Under the oscillating electromagnetic field [26], electron transfer and transition occur on the SiCnws, which improves the conductive loss of the composites. Finally, SiCnws and ceramic composite materials form a large number of heterogeneous interfaces, and a large number of electrons will accumulate at the interfaces with different conductivity on both sides. Under the oscillating electromagnetic field, interface polarization will be induced, thereby enhancing the dielectric loss [23]. The synergistic effect of these dissipation mechanisms improves the electromagnetic wave absorption properties of the composites.

## 4. Conclusions

In this paper, SiCnws were grown in situ on SiC porous ceramic materials by the sol–gel and carbothermal reduction method. The results show that the electromagnetic wave absorption performance can be effectively improved by growing SiCnws in SiC porous ceramics. The high specific surface area of SiCnws and the three-dimensional network structure formed with porous ceramics optimize the electromagnetic wave absorption interface of porous ceramics, enhance the impedance matching ability, establish the electromagnetic wave attenuation path, and jointly promote the electromagnetic wave absorption performance. The prepared composite with a C-to-Si ratio of 1:1 shows excellent electromagnetic wave absorption properties. At a thickness of 2.30 mm, the RLmin is −56.95 dB with an EAB of 1.85 GHz, and the optimal EAB of 4.01 GHz is at 2.59 mm. Therefore, the in situ growth of SiCnws makes SiC porous ceramics have an adjustable microstructure and dielectric properties, which will be a potential high-performance microwave-absorbing material in the future.

## Figures and Tables

**Table 1:** Porosity of SiC porous ceramics.

Sample	Dry Weight (*m*_1_)	Wet Weight (*m*_3_)	Suspended Weight (*m*_2_)	Porosity
1	5.61	6.36	3.85	29.9%
2	5.47	6.20	3.75	29.8%
3	5.58	6.34	3.83	30.3%

## Data Availability

The original contributions presented in the study are included in the article, and further inquiries can be directed to the corresponding authors.
